# Peripheral compressing artifacts in brain tissue from stereotactic biopsy with side-cutting biopsy needle: a pitfall for adequate glioma grading 

**DOI:** 10.5414/NP300404

**Published:** 2011-10-18

**Authors:** S.H. Kim, W.S. Chang, J.P. Kim, Y.K. Minn, J. Choi, J.W. Chang, T.S. Kim, Y.G. Park, J.H. Chang

**Affiliations:** 1 Department of Pathology,; 2 Department of Neurosurgery,; 3 Neuro-Oncology Clinic,; 4 Brain Research Institute, Yonsei University College of Medicine, Seoul,; 5 Department of Neurology, Hallym University, Chuncheon,; 6 Department of Pathology, Yonsei University College of Medicine, Seoul, Republic of Korea

**Keywords:** stereotactic biopsy, brain tumor, pathology, glioma, artifact

## Abstract

Aims: The stereotactic brain biopsy is an essential diagnostic procedure in modern neurologic patient management. A side-cutting biopsy needle is one of the most widely used needle types. Recently we found a characteristic tissue artifact named “peripheral compressing artifact” in the brain tissues biopsied using a side-cutting needle of Leksell’s system. We investigate prevalence, possible cause and its clinical implication of this type of artifact. Materials and methods: We examined the biopsies from 80 patients (44 cases of gliomas, 13 lymphomas, 7 germ cell tumors, 2 other tumors, 1 metastatic carcinoma, 4 non-tumorous conditions such as demyelinating disease and 8 non-diagnostic) in the stereotactic biopsy group with a suspected brain tumor, who underwent a stereotactic brain biopsy using side-cutting needle of Leksell’s system. We also evaluated 16 cases of open brain biopsies without Leksell’s system as a control group. Results: The artifact is a semi-circular or band-like tissue compression in the periphery of the biopsied tissue. This artifact was found in 30 (37.5%) out of 80 cases and 57 (11.9%) out of 477 biopsied pieces. It might be produced during rotating of the inner cannula of the biopsy needle. Histologically, it might be misinterpreted as “hypercellular”, “spindle”, “well circumscribed”, or rarely as “pseudopalisading” especially in glioma. Conclusions: Awareness of this artifact would help making the appropriate pathological diagnosis for glioma.

## Introduction 

Stereotactic brain biopsy is one of essential diagnostic procedures in modern neurologic patient managements [1, 2, 3, 4, 5]. The effectiveness, reliability, diagnostic yield, limitations and risks of stereotactic brain biopsy have been well evaluated [1, 2, 6]. Several stereotactic frames and apparatus have been used and a side-cutting biopsy needle is one of the most widely used biopsy needle [7, 8, 9]. 

There are variable macroscopic and microscopic artifacts (such as the Cavitron ultrasound surgical aspirator (CUSA) artifact, artifact from foreign materials, autopsy artifacts, etc.) from the brain tissue processing and evaluation [10]. However tissue artifacts of stereotactic biopsy have not been well described. Recently, we encountered a specific tissue artifact, named “peripheral compressing artifact” in case of using a side-cutting needle for biopsy. Herein, we investigate prevalence, possible cause and the clinical implication of this type of artifact. 

## Materials and methods 

**Figure 1.**
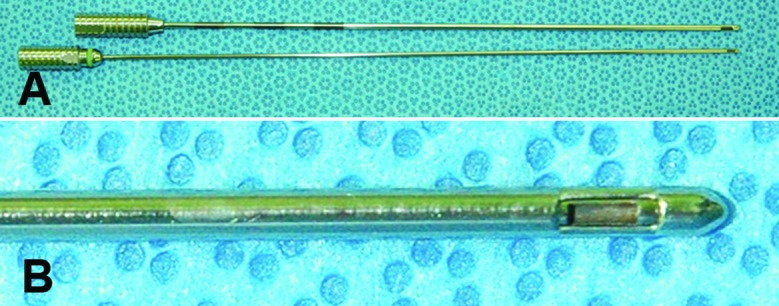
The side-cutting type brain biopsy needle of Leksell’s system. The outer (top of A) and inner (bottom of A) cannulae are seen. After assembly, both the side windows, through which tissue is aspirated, are aligned (B).

The cases of stereotactic brain biopsy performed between January 2005 and February 2011 using Leksell’s system were retrieved from the Pathology Department archives of our institution. The cases of frozen section biopsy were excluded because of the frozen section artifact. We enrolled 80 patients with a suspected brain tumor, who underwent a stereotactic brain biopsy using a side-cutting needle of Leksell’s system (Figure 1) with head frame (Elekta, Stockholm, Sweden). We also evaluated 16 cases of open brain biopsies without Leksell’s system as a control group. 

All patients underwent a stereotactic brain biopsy in a usual manner. The neurosurgeon advanced a brain biopsy needle with closed side opening toward the target of the lesion with MRI guidance. The side-cutting type brain biopsy needle consists of inner and outer cannulae, both of which contain a side window. When the needle tip reached the target, the surgeon opened the window by aligning the window of both cannulae. Through this opening, a piece of the lesion was aspirated by negative vacuum pressure. Then surgeon rapidly rotated the inner cannula of needle with constantly holding outer cannula to close the window, and a small piece of material was resected within the inner cannula in a guillotine action. Finally, the surgeon withdrew the inner cannula and collected the tissue samples. Next, routine histological processing was done according to conventional protocol. At the time of open brain biopsy, the neurosurgeon used “Yasargil bayonet tumor grasping forceps” (Aesculap, Tuttlingen, Germany). 

**Figure 2.**
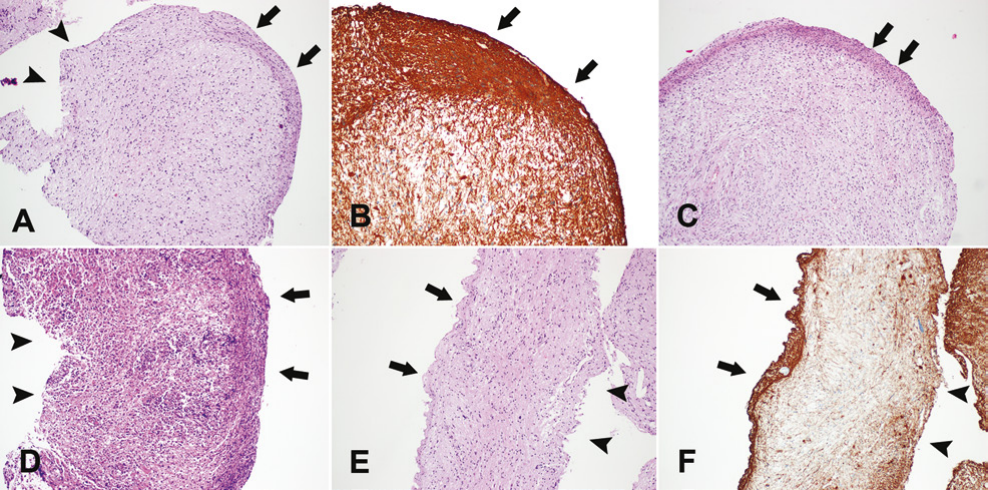
The peripheral compressing artifacts (arrows). Semi-circular (A, B, C) or band-like (D, E, F) tissue compression are observed (A, C, D, E: H&E × 100). The compressing artifacts are accentuated by the GFAP immunohistochemical staining (B: GFAP × 200, F: GFAP × 100). The opposite side (arrow heads) of the artifact does not show the compressing artifact (A, D, E, F). A, B and C are gliomas. D is primary central nervous system lymphoma. E and F are non-neoplastic glial tissues.

**Figure 3.**
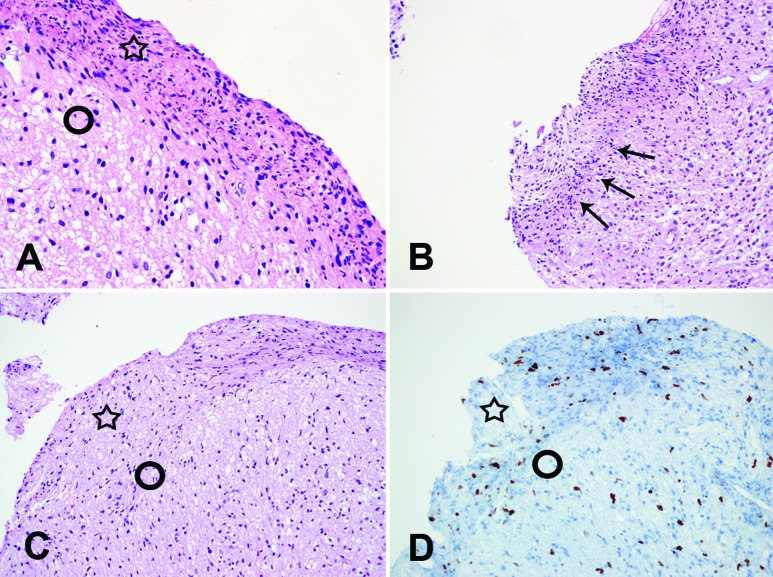
High power view of the compressing artifacts. The area of the compressing artifact (open star) shows more hypercellular and spindle effects than the area of no artifact (open circle) (A: H&E × 400). The compressing area (arrows) looks like “pseudopalisading” (B: H&E × 200). The compressing artifact area (open star) also looks like higher Ki-67 immunopositivity than no artifact area (open circle) (C: H&E × 200, D: Ki-67 × 200).

Two pathologists reviewed the pathologic diagnosis of the slides. They counted the numbers of biopsied pieces in each case and evaluated the existence of the peripheral compressing artifact. We defined the “peripheral compressing artifact” as semi-circular or band-like tissue compression in the periphery of biopsy samples (Figure 2, Figure 3). The severity of tissue compression was arbitrarily graded as “mild” (compressing thickness of less than 50 µm), “moderate” (compressing thickness of 50 – 100 µm), and “marked” (compressing thickness of more than 100 µm). The presence and severity of the tissue artifact were agreed upon by the two pathologists (SH Kim and J Choi). 

## Results 

The mean ages in the stereotactic biopsy group and the control group were 46.24 (11 – 78) and 51.94 (1 – 79) years, respectively. There were 44 cases of gliomas, 13 lymphomas, 7 germ cell tumors, 2 other tumors, 1 metastatic carcinoma, 4 non-tumorous conditions such as demyelinating disease and 8 non-diagnostic in the stereotactic biopsy group. All control group cases were gliomas. The total numbers of biopsy samples in the stereotactic biopsy and control groups were 477 and 87 samples, respectively. The mean numbers of biopsy samples were 5.96 (1 – 14) and 5.44 (1 – 15) per case. 

Among the 477 samples of stereotactically biopsied tissues, we found a “peripheral compressing artifact” in 57 samples (11.9%). The severities of peripheral folding were “mild” in 21 samples, “moderate” in 28 samples, and “marked’ in 8 samples. However, we found the “peripheral compressing artifacts” in 30 cases among a total of 80 cases (37.5%). When we counted the maximum severity, 12 (15.0%) and 11 (13.8%), and 7 (8.8%) cases showed “mild” and “moderate”, and “marked”, respectively. Among 30 cases that showed “peripheral compressing artifacts”, 19 cases were gliomas (Figure 2A, B, C), 3 cases were lymphomas (Figure 2D), 3 cases were germ cell tumors and 5 cases were non-diagnostic or non-neoplastic glial tissues (Figure 2E, F). In 19 cases of gliomas, 2 cases had been over-graded at first microscopic examinations. They had been mistakenly diagnosed as “anaplastic astrocytoma (Figure 2A, B)” and “suspicious of glioblastoma (Figure 3B)”, respectively. With the consideration of these artifacts, clinico-radiologic correlations and further histologic examinations such as serial sections, we could determine adequate histologic grading properly. In addition, 11 cases showed hemorrhage in the biopsied tissue. There was no correlation between “peripheral compressing artifacts” and hemorrhage (data not shown). 

There were no “peripheral folding artifacts” in the control cases. However, we observed other tissue artifacts such as pinching in the control cases. 

## Discussion 

The stereotactic biopsy has become one of the standard diagnostic tools for neurosurgical management although there were some limitations [1, 2, 6] such as “small tissue samples”, “procedure complications” and “targeting errors”. Beside these limitations, the technique of a stereotactic biopsy itself could cause some histologic artifacts [11]. However, the histologic artifacts of stereotactic brain biopsies which might influence pathologic diagnosis have never been evaluated. 

The artifact which we reported in this study is a semi-circular or band-like tissue compression in the periphery of the biopsied tissue. This artifact can be observed only when a side-cutting type biopsy needle is used. We presume that this tissue artifact might be caused by the mechanics of the side cutting needle used. As previously mentioned, a side-cutting biopsy needle consists of inner and outer cannulae and in the biopsy procedure, and during the biopsy procedure, a neurosurgeon rotated the inner cannula to close the side window and to cut the tissue. We thought that this artifact might occur during rotating the inner cannula, especially when the neurosurgeon rotated slowly. The artifact’s semi-circular or band-like shape, rather than a whole-circular shape (Figure 2A, B, C, D, E, F) support this possibility. The opposite side of the artifact did not show the same artifact (Figure 2A, D, E, F). The “peripheral compressing artifact” was accentuated with GFAP immunohistochemistry (Figure 2B, F). 

As mentioned in “Results”, we started this project since we have experienced 2 cases of biopsy that have been nearly diagnosed as higher grade glioma (diffuse astrocytoma to anaplastic astrocytoma and anaplastic astrocytoma to glioblastoma, respectively). Because of the compression, the border between the non-compressed portion and artifact of biopsied tissue is well demarcated (Figure 3A). In addition, glial cells in the compression area could look hypercellular and spindle (Figure 3A, C). We thought that these histologic features caused mis-grading of pathologic diagnosis of glioma. The clear demarcation of the border could be mistaken for a “circumscribed glial tumor such as pilocytic astrocytoma”. Also the features of hypercellular or spindle might be overdiagnosed or misinterpreted as “high grade glioma” or “sarcoma component of gliosarcoma”, respectively. A proliferating index such as Ki-67 could be mistakenly judged higher because of tissue compression in this area (Figure 3C, D). In addition, this artifact may on rare occasions assume a similar histologic finding (Figure 3B) called “pseudopalisading”, a hallmark of glioblastoma. However, the diagnoses of the other tumors (e.g., lymphoma: Figure 2F) and lesions (e.g., non-neoplastic glial tissue: Figure 2D, E) were not influenced by this artifact. 

In conclusion, in case of stereotactic biopsies using a side-cutting needle, “peripheral compressing artifacts” can be observed, and it might lead to misdiagnosis of glioma in term of subtyping or grading. Awareness of this artifact would help making the appropriate pathological diagnosis for glioma. 

## Acknowledgment 

This work was supported by the Mid-career Researcher Program and the Basic Science Research Program through a National Research Foundation grant funded by Korean Ministry of Education, Science & Technology to Jong Hee Chang (No. R01-2008-20545-0) and Se Hoon Kim (2010-0021092). 

The first author (SH Kim) thanks Dr. Gregory N. Fuller (MD Anderson Cancer Center, Houston, TX, USA) for his inspiring, passionate teaching.
